# Supplementary material for: Hydrothermal alteration of andesitic lava domes can lead to explosive volcanic behaviour
Source: Nat Commun. 2019 Nov 7;10:5063. doi: 10.1038/s41467-019-13102-8 (PMC6838104; doi:10.1038/s41467-019-13102-8)
Supplement: Supplementary file 1 — Supplementary Information [file 41467_2019_13102_MOESM1_ESM.docx]

Supplementary Information for

Hydrothermal alteration of andesite lava domes can lead to explosive volcanic behaviour

By Heap et al.

**Supplementary figures**

**
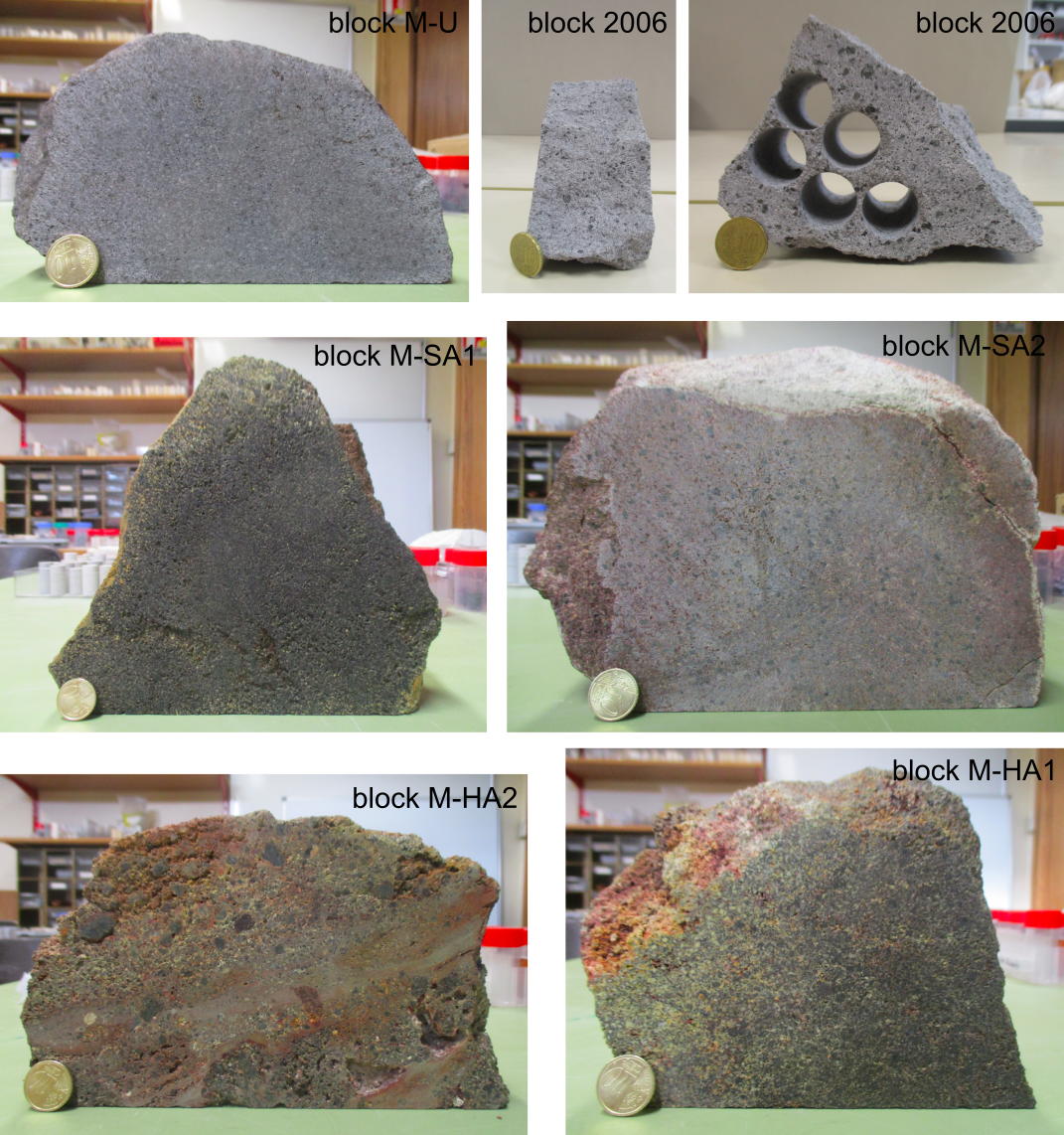
**

**Supplementary Figure 1.** Photographs of the six blocks collected from the summit of Merapi (Indonesia) for this study.


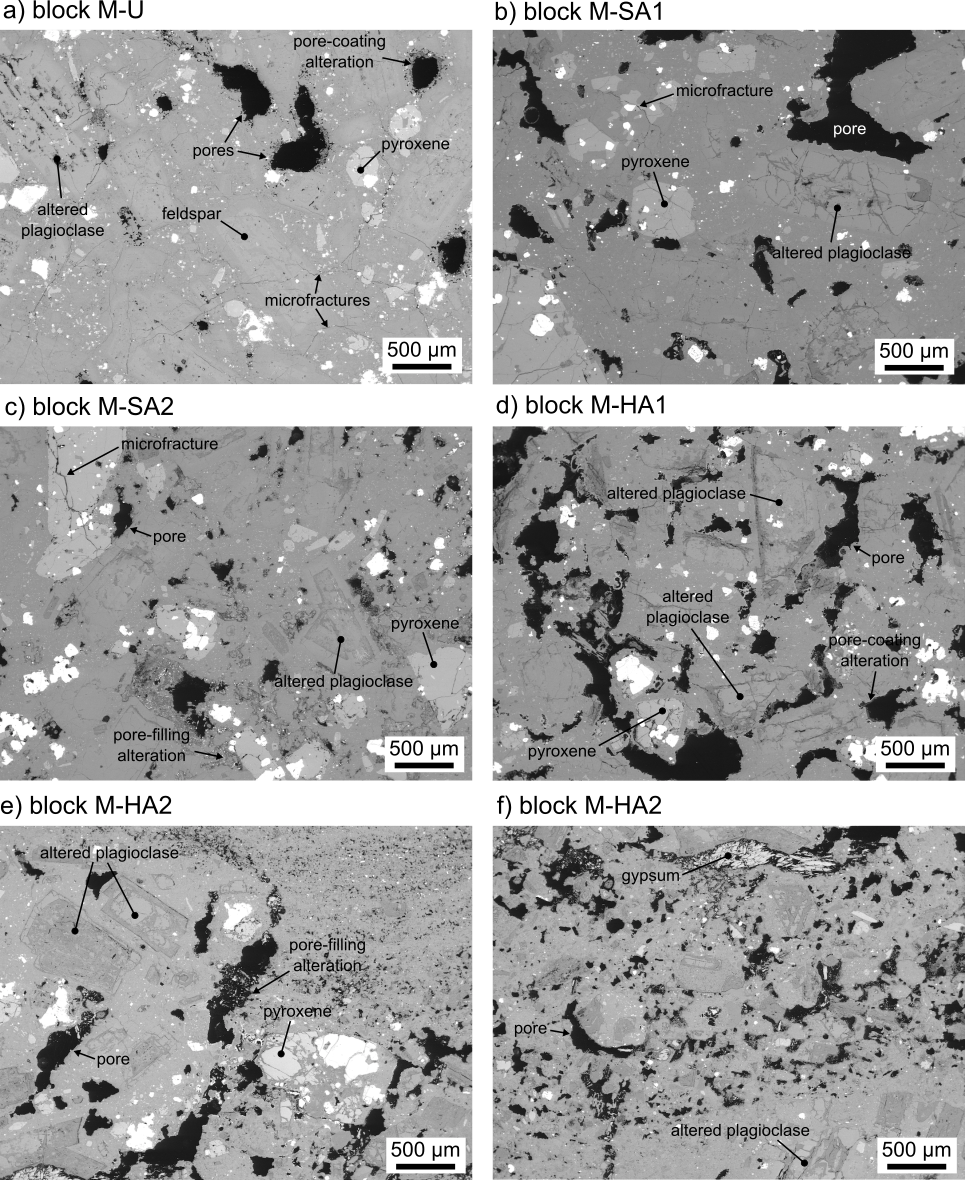


**Supplementary Figure 2.** The microstructure of variably altered Merapi dome rocks. Backscattered scanning electron microscope images (SEM) of each of the five main blocks collected for this study. Key features are labelled on the images. (a) SEM image of block M-U. (b) SEM image of block M-SA1. (c) SEM image of block M-SA2. (d) SEM image of block M-HA1. (e) SEM image of block M-HA2. (f) SEM image of block M-HA2.


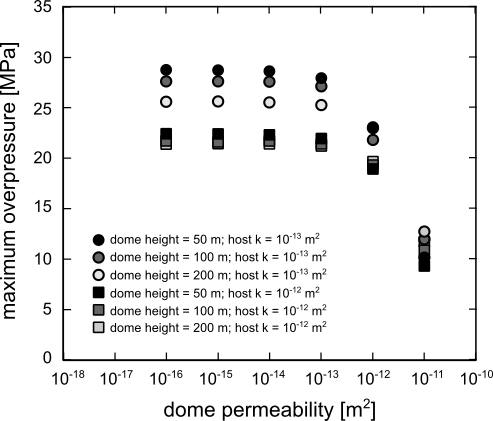


**Supplementary Figure 3.** Results from the COMSOL Multiphysics V4.3 numerical modelling. Maximum overpressure within the lava dome (for domes of different height and for different edifice permeabilities) as a function of lava dome permeability. See main manuscript text for details.


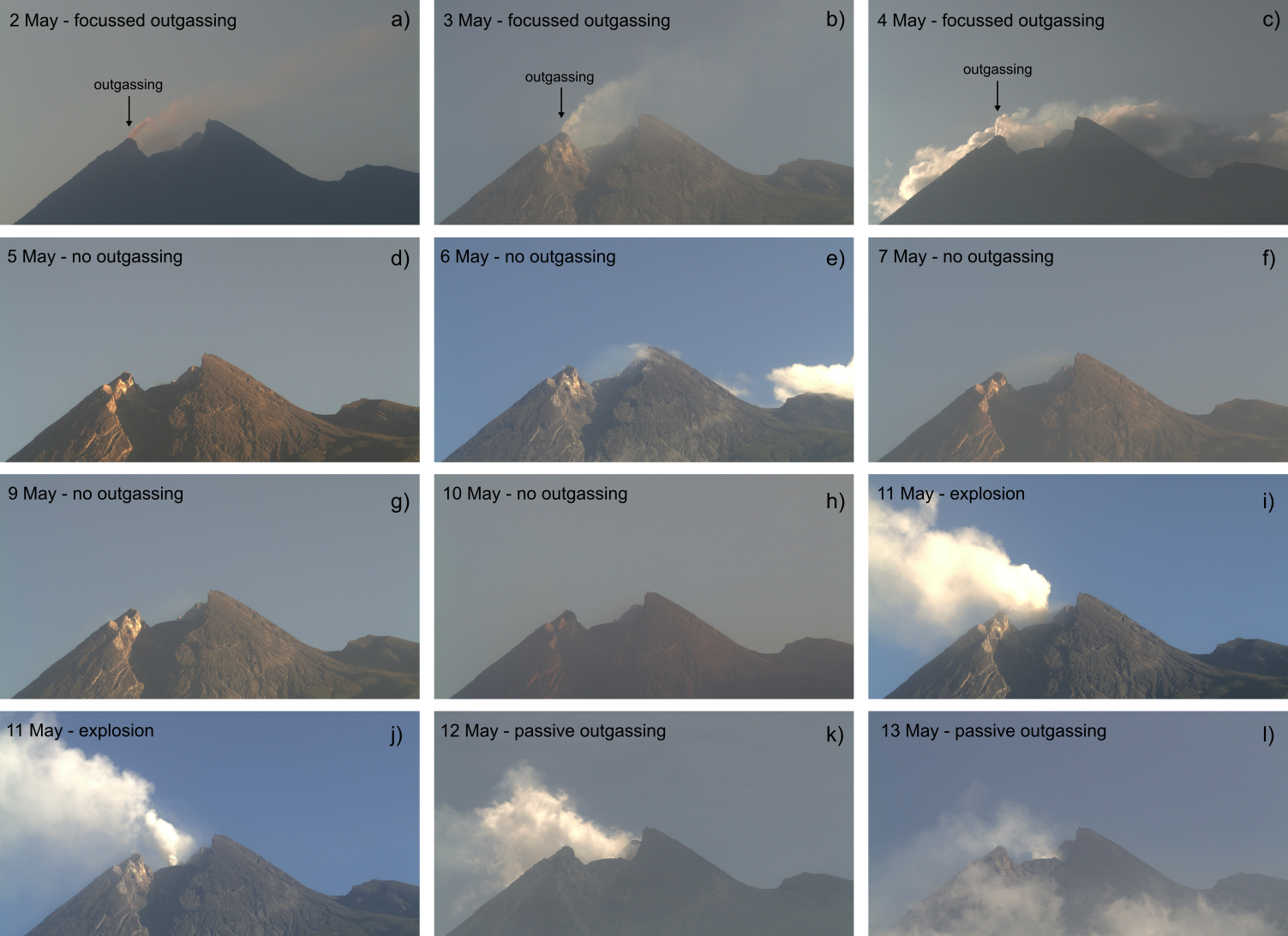


**Supplementary Figure 4**. Representative time-lapse photographs of the dome at Merapi volcano before, during, and after the 11 May 2018 explosion (no images were available for 8 May). Panels (a-c) show focussed outgassing. Panels (d-h) show no outgassing. Panels (i) and (j) show the 11 May explosion. Panels (k) and (l) show passing outgassing.

**Supplementary table**

| **Dome height (m)** | **Equivalent permeability (m^2^)** | | | **Maximum overpressure (MPa)** |
| --- | --- | --- | --- | --- |
|  | Magma-filled conduit | Edifice | Dome |  |
| 50 | 10^-10^ | 10^-12^ | 10^-11^ | 9.35 |
| 50 | 10^-10^ | 10^-12^ | 10^-12^ | 18.87 |
| 50 | 10^-10^ | 10^-12^ | 10^-13^ | 21.94 |
| 50 | 10^-10^ | 10^-12^ | 10^-14^ | 22.36 |
| 50 | 10^-10^ | 10^-12^ | 10^-15^ | 22.40 |
| 50 | 10^-10^ | 10^-12^ | 10^-16^ | 22.41 |
| 50 | 10^-10^ | 10^-13^ | 10^-11^ | 10.2 |
| 50 | 10^-10^ | 10^-13^ | 10^-12^ | 23.12 |
| 50 | 10^-10^ | 10^-13^ | 10^-13^ | 27.93 |
| 50 | 10^-10^ | 10^-13^ | 10^-14^ | 28.64 |
| 50 | 10^-10^ | 10^-13^ | 10^-15^ | 28.73 |
| 50 | 10^-10^ | 10^-13^ | 10^-16^ | 28.73 |
| 100 | 10^-10^ | 10^-12^ | 10^-11^ | 10.86 |
| 100 | 10^-10^ | 10^-12^ | 10^-12^ | 19.35 |
| 100 | 10^-10^ | 10^-12^ | 10^-13^ | 21.38 |
| 100 | 10^-10^ | 10^-12^ | 10^-14^ | 21.63 |
| 100 | 10^-10^ | 10^-12^ | 10^-15^ | 21.65 |
| 100 | 10^-10^ | 10^-12^ | 10^-16^ | 21.65 |
| 100 | 10^-10^ | 10^-13^ | 10^-11^ | 11.96 |
| 100 | 10^-10^ | 10^-13^ | 10^-12^ | 21.83 |
| 100 | 10^-10^ | 10^-13^ | 10^-13^ | 27.14 |
| 100 | 10^-10^ | 10^-13^ | 10^-14^ | 27.57 |
| 100 | 10^-10^ | 10^-13^ | 10^-15^ | 27.62 |
| 100 | 10^-10^ | 10^-13^ | 10^-16^ | 27.62 |
| 200 | 10^-10^ | 10^-12^ | 10^-11^ | 11.77 |
| 200 | 10^-10^ | 10^-12^ | 10^-12^ | 19.63 |
| 200 | 10^-10^ | 10^-12^ | 10^-13^ | 21.24 |
| 200 | 10^-10^ | 10^-12^ | 10^-14^ | 21.42 |
| 200 | 10^-10^ | 10^-12^ | 10^-15^ | 21.44 |
| 200 | 10^-10^ | 10^-12^ | 10^-16^ | 21.45 |
| 200 | 10^-10^ | 10^-13^ | 10^-11^ | 12.74 |
| 200 | 10^-10^ | 10^-13^ | 10^-12^ | 23.03 |
| 200 | 10^-10^ | 10^-13^ | 10^-13^ | 25.30 |
| 200 | 10^-10^ | 10^-13^ | 10^-14^ | 25.58 |
| 200 | 10^-10^ | 10^-13^ | 10^-15^ | 25.61 |
| 200 | 10^-10^ | 10^-13^ | 10^-16^ | 25.61 |

**Supplementary Table 1**. Results from the COMSOL Multiphysics V4.3 numerical modelling. The maximum overpressure within the lava dome increases as the permeability of the lava dome decreases from 10^-11^ to 10^-16^ m^2^. See main manuscript text for details.
